# Editorial: Challenges and progressions in herbal genomics

**DOI:** 10.3389/fpls.2023.1273769

**Published:** 2023-09-06

**Authors:** Yinghao Cao, Zemin Ning, Yiqiang Zhao

**Affiliations:** ^1^ Center for Bioinformatics, Institute of Basic Medical Sciences, Chinese Academy of Medical Sciences & School of Basic Medicine, Peking Union Medical College, Beijing, China; ^2^ The Wellcome Sanger Institute, Wellcome Genome Campus, Hinxton, Cambridge, United Kingdom; ^3^ College of Biological Sciences, China Agricultural University, Beijing, China

**Keywords:** herbal genomics, third-generation sequencing, genome assembly, medicinal plants, molecular identification

Traditional herbal medicines have been used for over 5,000 years and inceasingly receiving attention from researchers in recent time. However, the lack of genetic information on medicinal plants hinders the progress and applications of such research. This Research Topic aims to provide a wide perspective to showcase the latest challenges and progressions in Herbal Genomics. There is a need for a collection of recent research in the herbal medicine filed as technological advances in genome sequencing are seen and also combined with increased analytical capabilities. For example, high-quality genome assemblies not only enable to understand the biological functions of genes in medicinal plants, but also could explore their roles in the synthesis of pharmacological components. As a supplement to nuclear genome, organelle genome is also an important genetic resource of medicinal plants. Molecular identification techniques represented by DNA molecular markers provide reliable technology for precise identification and quality control of medicinal plants. In addition to herbal genome resources, the construction of medicinal plant sequence databases, the development of bioinformatics tools and pipelines have become bottlenecks in current medicinal plant research. The Research Topic provided a wide range of content on herbal genomics, including genome assembly, gene expression for biosynthetic pathway, phylogenetic analysis, and a summarized application of three generation sequencing technologies used in herbal genomics.

One major aspect of herbal genomics is to assemble high-quality genomes of medicinal plants for the study of the biological and pharmacological research. The widespread application of next-generation sequencing (NGS) and the emergence of third-generation sequencing (TGS) technology have facilitated the rapid development in this field. Using Pacbio long reads complemented with Hi-C technologies, Yin et al. assembled the *Curcuma longa* genome into 21 pseudochromosomes with a total length of 1.11 Gb and scaffold N50 of 50.12 Mb. Contraction analysis of gene families showed transcription factors, phytohormone signaling, and plant-pathogen interactions associated genes in adaptation to harsh environments. Moreover, candidate genes related to phytohormone signaling and carbohydrate metabolic responses were analyzed to associate with the induction of tuber formation. The work provided insight into tuber formation and curcumin biosynthesis in turmeric. Dong et al. reported a chromosome-level genome assembly of *Curcuma alismatifolia* with the genome size of 991.3 Mb and a scaffold N50 of 56.7 Mb. Nine highly expressed genes were identified by transcriptome analysis, encoding for six enzymes downstream of the anthocyanin biosynthetic pathway. In addition, they also found the dispersal and tandem duplications of terpenoid biosynthesis genes. The work could accelerate the selective breeding of *C. alismatifolia* varieties with higher ornamental and medicinal value. Fu et al. assembled two high-quality reference genome sequences which provide insight into the evolution of the *Cannaceae* family. The comparative analysis of the genomes of *C. edulis* and *C. indica* showed that genes in the starch and sucrose metabolic pathways were replicated with a remarkable level in *C. edulis*. Besides, they performed a GWAS analysis which revealed the location of key genes related to leaf color and morphology.

Organelle genomes, especially the chloroplast (cp) genomes, as a supplement to nuclear genomes, contain substantial information of evolution for medicinal plants. This information may have potential applications in molecular identification and quality control of medicinal plants. Zhou et al. sequenced and assembled seven chloroplast genomes of *Isodon rubescens* and found five highly variable regions which could be used as some potential molecular markers for identifying *Isodon rubescens* from its contaminants. Shi et al. did a comparative analysis of medicinal plant *Polygonatum kingianum* with related verticillium leaf type based on nine chloroplast genomes and found eleven divergent hotspot regions may provide potential molecular markers for the identification of the *Polygonatum* species with verticillate leaf types.

Small noncoding RNAs, such as microRNAs and siRNAs, which may be transmitted between organisms of the same species and of different species. In 2012, a surprising discovery was reported that plant miR168a could specifically regulate expression levels of the mammalian gene LDLRAP1, which confirmed that, plant diet miRNAs could transfer to mammalian tissues and affect expression of discreted targets (Zhang et al., 2012). Huang demonstrated that hundreds of thousands of small RNAs were detected in each herb after sequencing RNAs extracted from ten TCM herbs with known anti-inflammatory properties (Huang et al., 2019). This may reflect that miRNAs of medicinal plant could be existed as potential RNA therapeutics that playing functional remedial roles in traditional medicines. Recently, a Bencao (herbal) small RNA (sRNA) Atlas was constructed with more than 10 million unique small RNAs extracted from 245 traditional Chinese Medicines (TCM) (Cao et al., 2023). The free and open Bencao small RNA Atlas is a resource to provide the scientific basis for herbal medicine mechanism of action and to accelerate the development of oligonucleotide drugs.

Sequencing is a powerful tool to facilitate the research of herbal genomics. In this Research Topic, Gao et al. gave a review on application of third-generation sequencing (TGS) to herbal genomics. The authors introduced two main TGS techniques, PacBio SMRT technology and Oxford Nanopore technology, and did a comparison between TGS and NGS to different scenes of herbal genomics. The authors described the applications on decoding whole genomes of medicinal plants including nuclear genomes and organelle genomes, revelation of transcriptomes and molecular identification. They discussed the advantages and challenges for TGS and NGS to herbal genomics. For constructing a complete chromosome-levels genome, TGS has the mainly advantages than next-generation sequencing (NGS) such as phase haplotypes and alleles, span repeat regions and large SVs. On contrast, due to the small size of the circular genome, NGS is a more suitable method for organelle genome sequencing when considering cost. In addition, the authors listed organelle genome assemblies obtained by TGS techniques.

In conclusion, herbal genomics, which would give an opportunity to investigate the chemical and biological analyses of complicated herbal products, is progressing steadly over the years fueled by the rapid development in sequencing technologies. Future work should be focused on genetic background, agricultural traits and the medical quality of most traditional herbs to promote the research and downstream applications.

## Author contributions

YC: Writing – original draft. ZN: Writing – review & editing. YZ: Writing – review & editing.
